# Prevention of sternal dehiscence with the Sternum External Fixation (Stern-E-Fix) corset – a randomized trial in 750 patients

**DOI:** 10.1186/1749-8090-7-85

**Published:** 2012-09-09

**Authors:** Lachmandath S Tewarie, Ares K Menon, Nima Hatam, Andrea Amerini, Ajay K Moza, Rüdiger Autschbach, Andreas Goetzenich

**Affiliations:** 1 Department of Cardiothoracic and Vascular Surgery, University Hospital RWTH Aachen, Pauwelsstrasse 30, 52074, Aachen, Germany

**Keywords:** Cardiac surgery, Sternal dehiscence, Mediastinitis, Sternum external fixation corset (Stern-E-Fix)

## Abstract

**Background:**

The main objective of this study will be to determine the effects of a new advanced sternum external fixation (Stern-E-Fix) corset on prevention of sternal instability and mediastinitis in high-risk patients.

**Methods:**

This prospective, randomized study (January 2009 – June 2011) comprised 750 male patients undergoing standard median sternotomy for cardiac procedures (78% CABG). Patients were divided in two randomized groups (A, n = 380: received a Stern-E-Fix corset postoperatively for 6 weeks and B, n = 370: control group received a standard elastic thorax bandage). In both groups, risk factors for sternal dehiscence and preoperative preparations were similar.

**Results:**

Wound infections occurred in n = 13 (3.42%) pts. in group A vs. n = 35 (9.46%) in group B. In group A, only 1 patient presented with sternal dehiscence vs. 22 pts. in group B. In all 22 patients, sternal rewiring followed by antibiotic therapy was needed. Mediastinitis related mortality was none in A versus two in B. Treatment failure in group B was more than five times higher than in A (p = 0.01); the mean length of stay in hospital was 12.5 ± 7.4 days (A) versus 18 ± 15.1 days (B) (p=0.002). Re-operation for sternal infection was 4 times higher in group B. Mean ventilation time was relatively longer in B (2.5 vs. 1.28 days) (p = 0.01). The mean follow-up period was 8 weeks (range 6 – 12 weeks).

**Conclusions:**

We demonstrated that using an external supportive sternal corset (Stern-E-Fix) yields a significantly better and effective prevention against development of sternal dehiscence and secondary sternal infection in high-risk poststernotomy patients.

## Background

Mechanical sternal dehiscence in post-sternotomy cardiac surgery patients is a devastating complication. Not only patients discomfort and pain are affected, it has also a huge impact on patients’ morbidity and even mortality, which increases hospital costs and social burden. Since introduction of median sternotomy by Milton (1897) [1], many authors suggested a growing diversity of sternal wiring techniques, from single wire to more modified “figure of eight”-wires and cable closure techniques, even dynamic fixation plates have been discussed. Still we are dealing with the same problems as a few decades before. Using other sternum fixation techniques with osteosynthesis plates, clamps and similar devices after primary sternotomy did not lead to a huge benefit. Although those devices are expensive and their application is time consuming, some authors suggest that they represent a good alternative [2-4] for secondary sternal fixation. Besides the aforementioned wiring techniques we need postoperative precautions to prevent sternal complications, as specific activity restrictions alone will not reduce the risk. The problem lies not only in the wiring techniques or postoperative sternal precautions but its causes are multifactorial. Since the last few decades, we are operating in a totally different patient population. Nowadays our patients are severe obese, diabetic, with obstructive pulmonary disease, are mostly smokers and old.

As we all know mediastinitis is a multifactorial disease with an incidence between 0.5 and 5%. The main independent risk factors are still: obesity, diabetes, smoking, COPD, use of pedicled internal thoracic artery and prolonged on-pump time [5-7].

Sternal wound infections are significantly related to levels of obesity (24 < BMI < 30). The length of stay in OR and ICU (ventilation time) is increased in patients with high extremes of BMI (> 30) [8-10]. High blood glucose levels are associated with a higher incidence of deep wound infection. Insulin-treated diabetes has a poorer midterm survival and higher incidence of reoperations for mediastinitis [10-12]. Another independent risk factor is the use of a pedicled internal thoracic artery (ITA). Using ITA is associated with 4-20-fold increase risk of sternal wound infection [13-18].

Worldwide, many external sternal stabilizers have been introduced, the success rates can be questioned. Each of those stabilizers has been described as a good conservative approach to stabilize the poststernotomy sternum and to prevent mechanical sternal dehiscence and deep sternal infection. In our opinion such external supportive sternal stabilizers must meet the following criteria: The device must be (1) functional without any thorax function impairment, (2) easy to use, (3) decrease poststernotomy discomfort and pain without restrictions in physical activity and (4) increase quality of life. Since January 2009, we are using an external sternal stabilizer, a Stern-E-Fix (SEF) corset (Fendel & Keuchen GmbH, Aachen Germany) in all multi-risk patients operated in our hospital.

The main objective of this study will be to determine the effects of an advanced sternum external fixation (Stern-E-Fix) corset on prevention of sternal instability and mediastinitis.

## Methods

This prospective, randomized study comprises 750 male patients undergoing cardiac surgery at our institute from January 2009 till June 2011. All patients underwent similar preoperative preparations for different cardiac procedures (78% CABG with pedicled IMA harvesting, 22% other cardiac procedures), using standard median sternotomy. Patients were divided in two randomized groups: in group A, n = 380 pts. postoperatively received a Stern-E-Fix corset. In group B, n = 370 pts. received a standard elastic thorax bandage for 6 weeks.

All patients with sternotomy were included following informed consent. The research was carried out in compliance with the Helsinki Declaration and approved by the local ethics committee. On the first postoperative day, all high-risk patients received an external sternal corset (Group A) or elastic bandage (Group B). All patients were evaluated on a daily base. During the hospitalization, patients were instructed on how to use the corset properly and advised to wear the device until six weeks after sternotomy. Sternal wound infections and mediastinitis were classified according to the guidelines of the Center for Disease Control and Prevention (CDC) [19] and according to the El Oakley and Wright classification [20] (Table 1). 

**Table 1:** Classification of mediastinitis according to Reida M. El Oakley, J.E.Wright (Dept. Card.Surg, Royal Brompton Hospital, London)

	
I	Mediastinitis presenting within 2 weeks after operation in the absence of risk factors
II	Mediastinitis in 2 – 6 weeks after operation in the absence of risk factors
	- A: Med type I in presence of one or more risk factors
	- B: Med type II in presence of one or more risk factors
IV	- A: Med type I, II, or III after one failed therapeutic trial
	- B: Med type I, II, or III after more than one failed trial
V	Mediastinitis presenting for the first time more than 6 weeks after operation

In case of diagnosed wound infection, appropriate antibiotics were administered based on culture and sensitivity results. Patients with mechanical sternal dehiscence without clinical evidence of wound infection were treated conservatively with SEF-corset (group A) or elastic bandage (group B). In some cases (1/380 (group A) and 22/370 (group B)) surgical rewiring was needed. When mediastinitis became evident, surgical debridement (necrotic tissue and steel wires) followed by temporary vacuum sealing (VAC) was performed. Definite sternal wound closure followed after sterile microbiological cultures were confirmed. Wound closure was accomplished by mobilizing vascularized subcutaneous tissue or pectoral muscle flaps.

To prevent non-compliance to our treatment regimen and precautions, we used a specifically validated questionnaire, which was completed in a mean follow up time of 8 (±3.6) weeks. In the first postoperative week patients were evaluated for pain relief and discomfort during coughing, functional disability and restrictions in daily life and quality of life. After discharge, the questionnaire was completed in sixth week by telephone interview.

### Stern-E-Fix corset

#### Fendel & Keuchen GmbH, Aachen Germany

**Figure 1:**
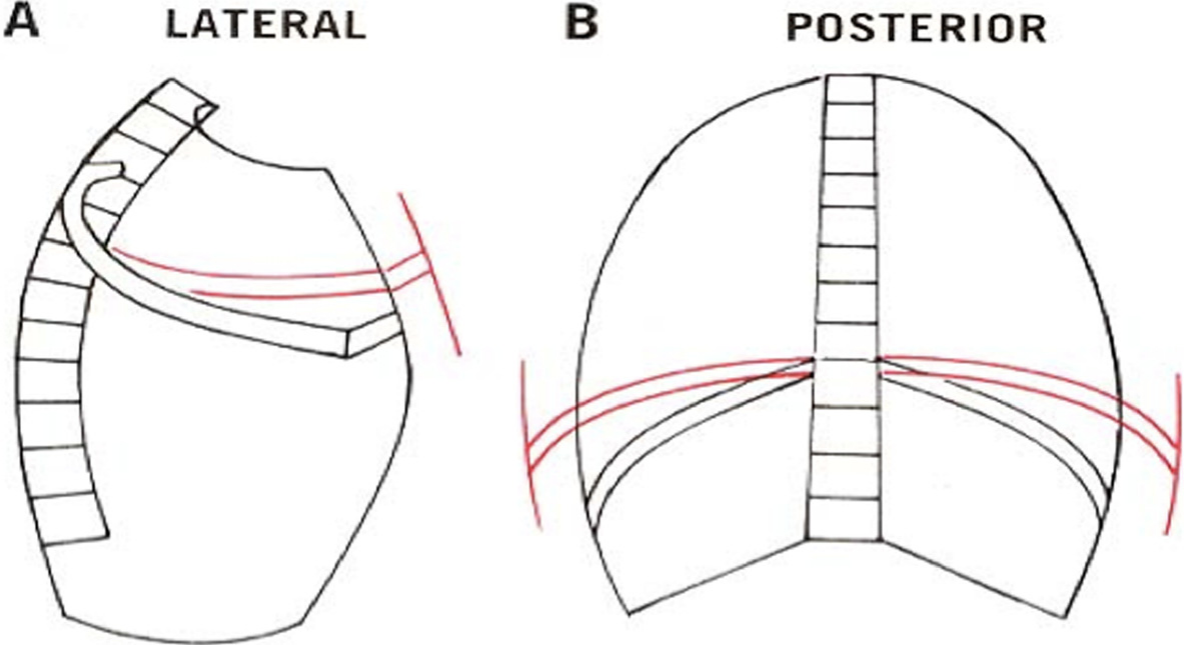
Physiological movement of upper and lower ribs during inspiration.

Physiology and pathophysiology of the Thorax:During breathing the rib cage moves upwards and outwards. The different orientations produce different arcs of rib motion. When the superior ribs elevate, their movement expands the ribcage in an anterior direction. Elevation of the inferior ribs expands the ribcage in a lateral direction (Figure 1). In this context, coughing can be described as a modified Valsalva maneuver. During vigorous coughing, intrathoracic pressures (up to 300 mmHg) and expiratory velocities (up to 28,000 cm/s or 500 miles/hour) lead to excessive thorax expansion. In a post- sternotomy thorax such forces can cause a variety of profound physically adverse effects that have the potential to lead to a significant increase in sternum instability, wound dehiscence and secondary to mediastinitis.

Design of the SEF-corset:Our concept was to create a symmetrical corset, which is moulded from the anatomical properties of the human thorax.

The following criteria were taken in consideration:

1 ease of use

2 functionality and prevention of excessive thorax expansion

3 Patients physical activities will not be restricted

4 preservation of lung function and auxiliary respiratory muscle movement

5 improvement in quality of life by pain reduction and provision of security

**Figure 2:**
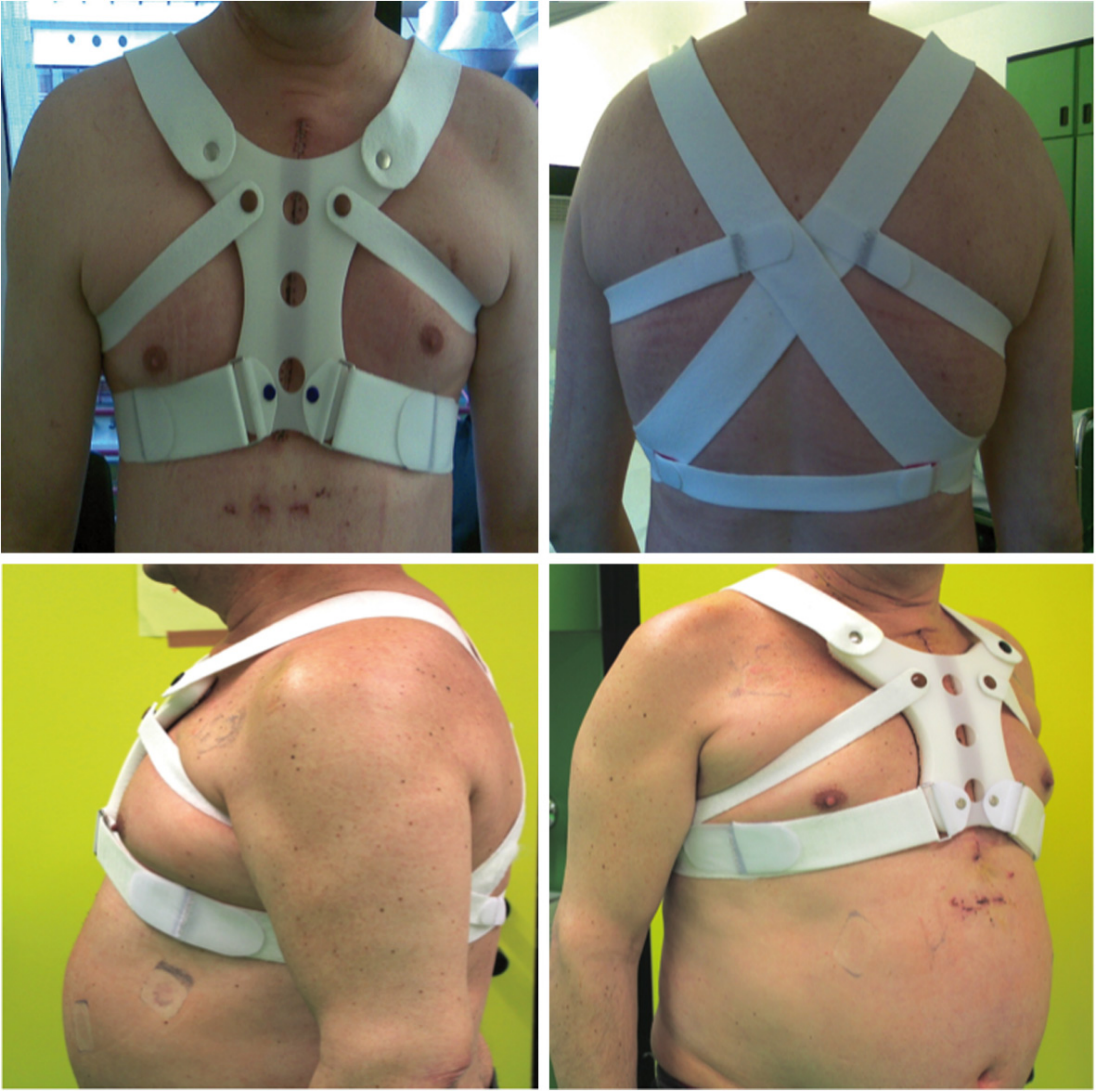
SEF-corset: in anterior -, posterior -, lateral - and anterolateral view.

The resulting SEF-corset (Figure 2) is divided in two main parts: The front plate is a strengthened plastic with elastic flexibility in the shape of the sternum. The contact surface is of smooth silicon and anti-allergic. This compound forms the main part of the SEF-corset. The front is connected with adhesive elastic bandages that follow the rib cage. Together with these, the superior adhesive bandages prevent excessive movement of the rib cage in an anterior direction. The inferior bandages prevent excessive rib cage movement in a lateral direction. Because of the elastic flexibility of the SEF-corset patients are not restricted during the postoperative rehabilitation.

### Statistics

Data analysis (Fisher square and students' t-test where appropriate) was performed with the SPSS software, version 19 (IBM, Chicago, IL, USA). Exact p- values are given, a p-value < 0.05 was considered statistically significant and is highlighted.

## Results

In both groups, risk factors for sternal dehiscence (age, body mass index, diabetes, smoking, COPD, IMA harvesting, Prolonged operation time, peripheral arterial disease, ventilation time etc.) were comparable. Incidence of renal failure was significantly higher in group B (14.9% versus 9.5%; p = 0.026). Additionally, in Group B, 35% of patients with renal failure also suffer an insulin-treated Diabetes mellitus as compared to only 15% in group A (Tables 2 and 3: patients demography).

**Table 2:** Description of preoperative clinical characteristics: BMI = Body mass index (kg/m2); COPD = chronic obstructive pulmonary disease; DM = diabetes mellitus; PAD = peripheral arterial disease; Log ES = log Euro score

**Risk Factors**	**A. Stern-E-Fix corset N = 380**	**B. Elastic Bandage N = 370**	***P-value***
Mean Age (years)	63.9 (± 10.9)	65.9 (±10.6)	0.518
BMI > 30	72.5%	74.2%	0.621
COPD	60.5%	59.6%	0.766
DM (I + II)	48.5%	45.8%	0.465
PAD	40%	38.3%	0.654
Renal Failure	9.5%	14.9%	0.026
Mean log Euro score	4.96% (± 4.3)	3,89% (± 4.9)	0.581

**Table 3:** Description of surgical technique: LITA = left internal thoracic artery; BITA = bilateral internal thoracic artery: OPCAB = off pump coronary artery bypass grafting; Beating heart = on pump beating heart; CPB = cardiopulmonary bypass; Others = other open heart surgery procedures

**Risk Factor**	**A. N = 380**	**B. N = 370**	***P-value***
Use of ITA	78%	79.9%	0.592
LITA	73%	78.9%	-
BITA	5%	1%	-
Others	22%	20.1%	0.531
OPCAB	11.2%	12.7%	0.576
Beating heart	11.3%	8.5%	0.181
CPB	77.5%	78.8%	0.724
Mean CPB time (min)	59.6 (±33.3)	58.2 (±36.9)	0.575
Mean operation time (min)	93.5 (±45.9)	86.7 (±54.5)	0.186

Pre-operative preparation and postoperative wound management were also similar in both groups. The sternal wiring technique was equal for both groups, 8 stainless steel (Ethicon) single wires or modified figure of eight. In both groups, the external corset and elastic bandage were used as soon as possible after sternotomy.

During hospitalization, patients in both groups were regularly evaluated for signs and symptoms of delayed wound healing or wound infections. Sternal instability is characterized by excessive sternal motion due to sternal non-union or fracture with the resultant pain and discomfort typically creating restrictions in the performance of activities of daily living. Deep sternal wound infections, or mediastinitis, is classified into four subtypes based on the time of first presentation, the presence or absence of risk factors, and whether previous attempts at treating the condition have failed [19,20]. The majority of patients with postoperative mediastinitis had polymicrobial infections. Sternal wound infection with Methicillin resistant staphylococcus (MRSA) occurred in 1 pt. (A) versus 3 pts. (B). Other microbiological findings were 74% Staphylococcus (Aureus or Epidermidis, MRSA included), 14% E.Coli, 6% Enterococcus, 4% Klebsiella and 2% Serratia marcescens**.**

**Figure 3:**
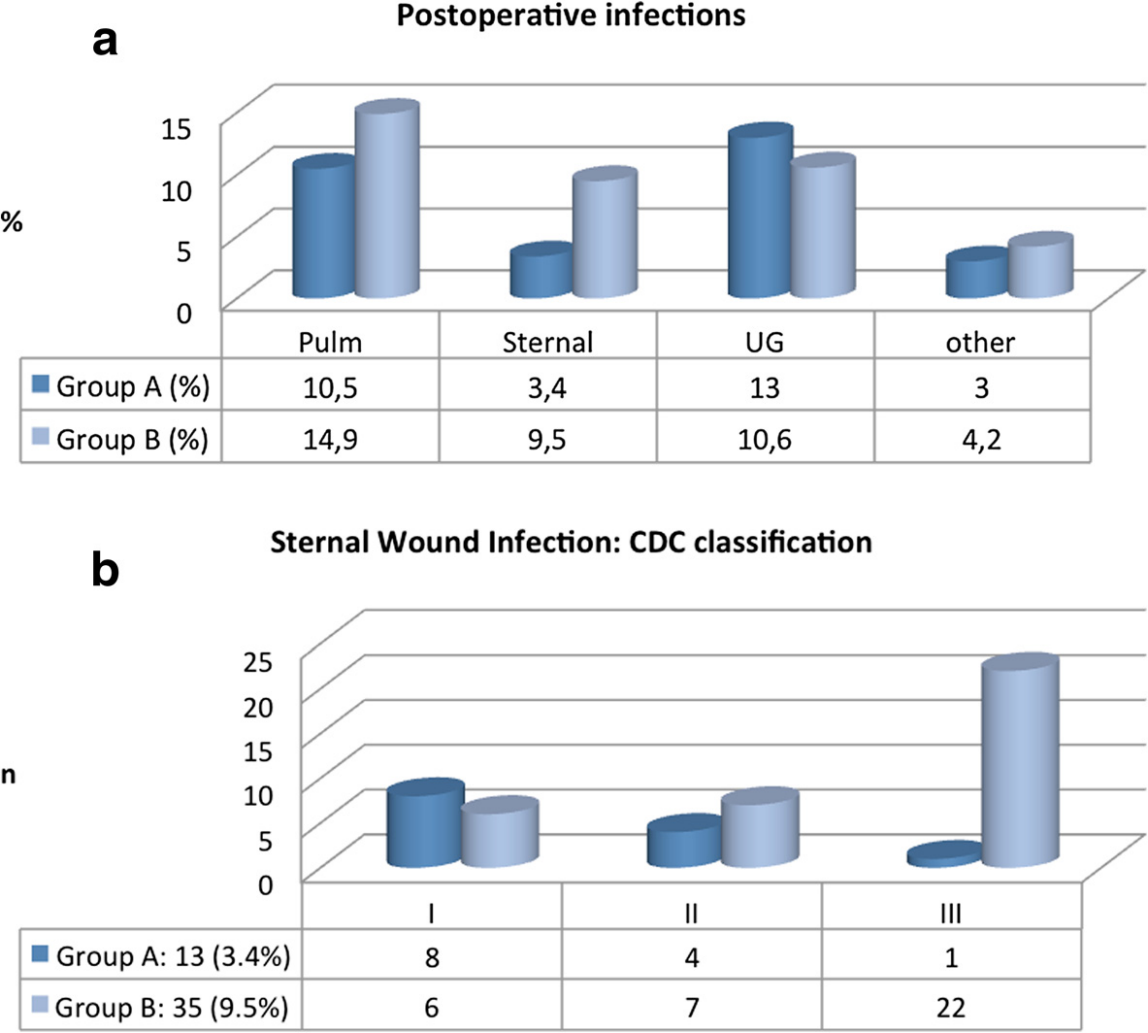
**a. Postoperative infections. **Description of postoperative infections: Pulm = pulmonary infections; Sternal = sternal infections according to center of disease control (CDC) classification; UG = urogenital infections; other = unspecified clinical infections. **b. Sternal wound infections.**

Postoperative data is summarized in Figures 3a and 3b. In group A, 13 (3.4%) patients developed sternal wound infections. 8 patients developed superficial (CDC I), 4 patients deep surgical wound infection (CDC II) and 1 pt. developed MRSA mediastinitis (CDC III). Sternal wound debridement and conservative therapy with antibiotics were needed. There was no mediastinitis related mortality. In group B, 35 (9.5%) patients developed sternal wound infection. 6 patients were categorized to CDC class I, 7 patients class II, 22 class III, accordingly.

**Figure 4:**
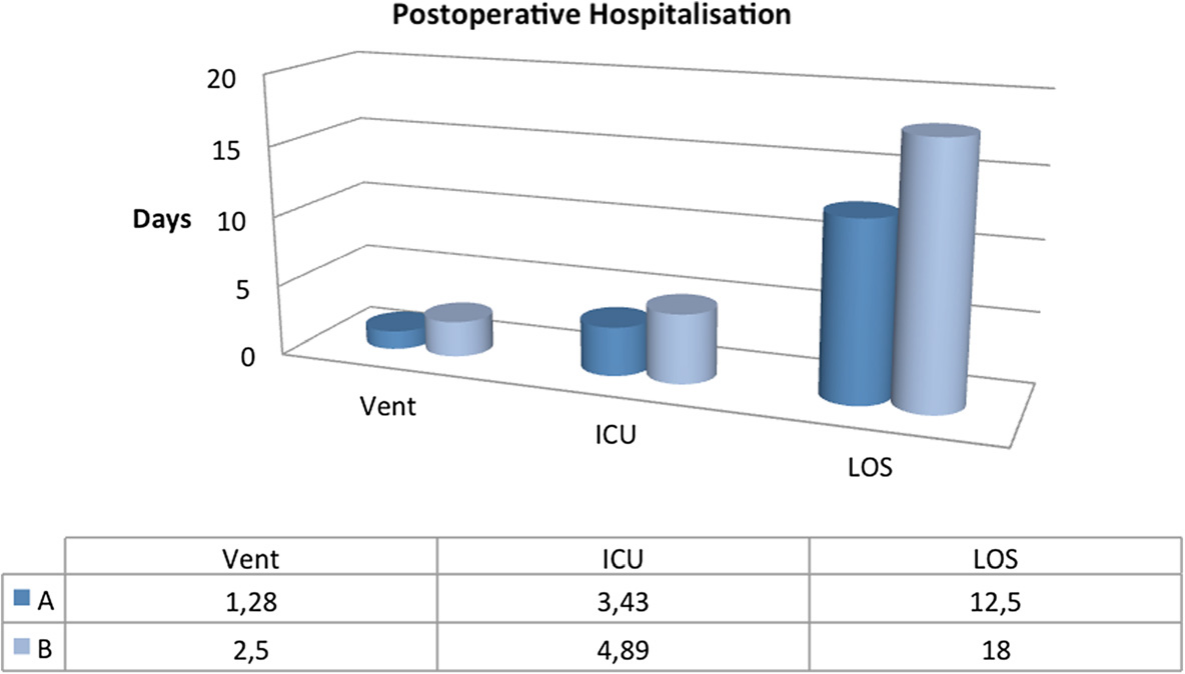
Description of postoperative clinical characteristics: Vent = ventilation time (p = 0.01); ICU = Length of stay on Intensive Care Unit (p = 0.01); LOS = total length of stay in hospital (0.002).

In group B, all 22 CDC III pts. developed partial or total sternal dehiscence (El Oakley class: 9 IIIa, 8 IIIb, 3 IVa resp. 2 IVb). In 19 patients sternal wires cut through sternal bone, in 3 pts sternal wire fracture occured. In all 22 patients, surgical treatment followed by antibiotic therapy was needed. There were two cases of mediastinitis related mortality; treatment failure was more than five times (p = 0.056) higher as compared to group A; The necessity of re-operation for sternal infection was 4 times higher in B versus A. Mean ventilation time was relatively longer in B (2.5 vs. 1.28 days, p = 0.01). In group A, one patient developed MRSA mediastinitis. The mean length of hospital stay was (B) 18 (±15.1) vs. 12.5 (±7.4) days in A (p = 0,002) (Figure 4). Relation to other infections was unclear and statistically not significant. Postoperative rehabilitation and mobilization was very effective in group A, because of increase in sternum stability, less discomfort and pain. The mean follow up period was 8 (±3.6) weeks.

In group B, there were two patients with chronic wound pain. In group A most patients (96%) were very pleased and satisfied with the advanced external sternal fixation corset.

## Discussion

**Figure 5:**
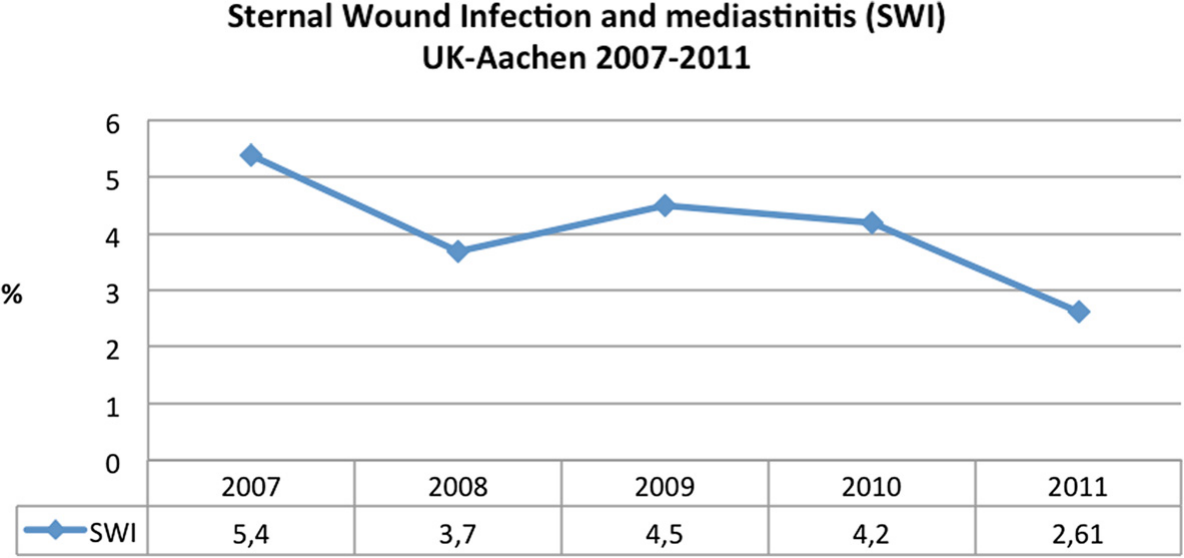
**Sternal wound infection and mediastinitis. **Decreasing trend in the last five years, in University Hospital Aachen, Germany. SWI = sternal wound infection and mediastinitis in poststernotomy patients.

Due to an aging population and increasing number of comorbidities, the operative risk has risen over the years. In the last decades, studies reported an improvement in cardiac surgery techniques, perioperative and postoperative management. As a result, despite the trend towards a worsening surgical risk profile, the combined morbidity and mortality rate remained unchanged [21,22]. The prevalence of multimorbid patients undergoing cardiac surgery is progressively increasing. The western population is steadily aging, the presence of comorbidities and life-threatening complications becomes more common. In our own institution we are currently operating more than 70% multimorbid patients yearly, which has increased in the past years. Off-pump cardiac surgery was performed in around 11% of our study group. We didn’t find an improvement in postoperative complications and significant reduction in poststernotomy sternal infections (p = 0.285). Use of the internal mammary artery (IMA) was standard in our studied group (80-90%). Only pedicled IMA was used. In our study, the overall sternal infection rate (6.4%, N = 750 / 2.5 years) could be considered low (2.6% /year) (Figure 5). 

Cohen et al. [23] evaluated the biomechanical property of three different Sternotomy closure techniques. He found that sternal separation occurred mostly at the xiphoid as a result of wires cutting through bone. Also, sternal distraction (2.0 mm) occurred with the least force in the lateral direction and the greatest force in the rostral-caudal direction with anterior-posterior force intermediate. In another investigation, greater separation occurred at the lower end of the sternum than the upper. Failure of the steel wire system usually involves the wire cutting into the bone by force, which results in a sternal nonunion and chronic dehiscence [23-27].

Other closure techniques include the basket weave formation by Robiscek or parasternal steel bands or Mersilene (Ethicon) ribbon was introduced to achieve wider force distribution at the lateral edge of the sternum. Ozaki et al. introduced a modified sternal plating technique, which effectively distributes force across the sternum. Despite a few advantages none of the aforementioned techniques has been widely adopted by the cardiac surgery community or in our institute as a standard sternal closure technique [21,22,28-30]. Given reasons against the use were: difficult handling, high costs, excessive corpus alienum use and the large size of some devices limits placement at lower intercostal spaces, especially at the xiphoid area which is most prone to wire migration.

Sternal dehiscence leads to discomfort, mediastinitis, osteomyelitis, and chronic sternal instability, and is associated with a 10% to 40% mortality rate worldwide [26]. In our study, two patients died due to mediastinitis. One patient died with systemic sepsis that resulted in multi organ failure. One patient died after a vacuum assisted cleaning system was used, which resulted in right ventricle wall rupture.

The human sternum is protected against stress forces because of its geometry and density. Following median sternotomy, several forces act on the sternum. Normal breathing and coughing stress the sternum through a combination of lateral displacement and transverse shear whereas longitudinal shear is applied to the sternum during skeletal movement, particularly when patients are using their arms to get in and out of bed. Using a SEF corset protects against those stress forces. The sternum shape of the SEF-front plate stabilizes the sternum during distractive forces (e.g. coughing and breathing) and decreases the stress on the sternum by distributing the forces over a larger area. Postoperative extreme physical sternal motion is very common during rehabilitation. Physical precautions and restrictions to prevent sternal complications are a challenging factor for every therapist. Restrictions in shoulder range of motion, lifting, reaching, dressing, exercise, driving, and a variety of other tasks have been reported [24,25,27]. There appears to be no consistency in the type or duration of restriction. More than 50% patients in group B developed sternal dehiscence after discharge from hospital or during the rehabilitation. Prevention of extreme physical sternal motion and intrathoracic sternal stress with a supportive external sternal instrument is needed in this rehabilitation period. Such a viable instrument against sternal stress forces was demonstrated in group A, with extremely low incidence of sternal dehiscence. The SEF-corset is not an instrument to prevent against soft tissue infection. The soft tissue infection (CDC class I and II) has a multifactorial cause. Last but not least the most important factor to prevent sternal complications is related to patients psychological behavior. The main concerns for patients with multimorbidity are loss of function, polypharmacy, a negative effect on their well-being and relationships, and difficulties with coordination of their care. The presence of such conditions lead to reduction in health-related quality of life, in the potential benefits of rehabilitation and contribute to organ complications and even mortality [26,31]. In our study group multimorbidity (COPD, age > 70 yrs., Diabetes, smoking, peripheral arterial disease) (70%) is very common and it has an impact on postoperative rehabilitation. Bitkover and associates concluded in a prospective computed tomography scan study of sternal healing after median sternotomy, that there was no sternal healing 3 months after operation and complete healing in only half of the patients by 6 months after operation. The method of sternal repair thus seems to be of great importance to long-term sternal stability [32,33]. In our study group we did not find any patients with sternal dehiscence after 12 weeks. Besides sternal precautions we did not recommended our patients further treatment with SEF-corset after 12 weeks.

The average cost of hospitalization of patients with wound infection is three times that of patients with an uncomplicated postoperative course. These excessive costs are primarily due to the associated high morbidity, prolonged hospital stay, and the need for repeated surgical procedures in these patients. We demonstrated that using a SEF-corset prevents against sternal dehiscence and mediastinitis, which results in reduction of length and cost of hospitalization.

## Conclusion

Mediastinitis is a devastating complication, which can lead to prolonged hospitalization, high hospital costs, high associated morbidity and even mortality. It is important to focus not only on efficient aseptic preoperative preparations and surgical techniques but even more on postoperative prevention techniques.

In the next decades, we will perform a growing number of sternotomies in high- risk patients. This phenomenon is the consequence of an aging population and an increase in comorbidity. We demonstrated in our study that using an external supportive sternal corset (Stern-E-Fix) yields a significantly better and effective prevention against development of sternal dehiscence and secondary sternal infection in high-risk patients. This growing patient population will likely benefit from such an effective supportive sternal corset instead of postoperative sternal precautions and restrictions alone.

## Competing interests

The authors declare that they have no competing interests.

## Authors’ contribution

All authors contributed to this manuscript, read and approved the final manuscript. LST designed the study. AjKM, NH, AA, ArKM and RA participated in the clinical proceedings and included patients to the study. LST and AG gathered the data, performed the statistics and wrote the manuscript. RA and AjKM corrected the manuscript.
